# Workplace interpersonal conflicts among the healthcare workers: Retrospective exploration from the institutional incident reporting system of a university-affiliated medical center

**DOI:** 10.1371/journal.pone.0171696

**Published:** 2017-02-06

**Authors:** Jih-Shuin Jerng, Szu-Fen Huang, Huey-Wen Liang, Li-Chin Chen, Chia-Kuei Lin, Hsiao-Fang Huang, Ming-Yuan Hsieh, Jui-Sheng Sun

**Affiliations:** 1 Center for Quality Management, National Taiwan University Hospital, Taipei, Taiwan; 2 Department of Internal Medicine, National Taiwan University Hospital, Taipei, Taiwan; 3 Department of Nursing, National Taiwan University Hospital, Taipei, Taiwan; 4 Department of Physical Medicine & Rehabilitation, National Taiwan University Hospital, Taipei, Taiwan; 5 Department of Orthopedic Surgery, National Taiwan University Hospital, Taipei, Taiwan; Duke University, UNITED STATES

## Abstract

**Objective:**

There have been concerns about the workplace interpersonal conflict (WIC) among healthcare workers. As healthcare organizations have applied the incident reporting system (IRS) widely for safety-related incidents, we proposed that this system might provide a channel to explore the WICs.

**Methods:**

We retrospectively reviewed the reports to the IRS from July 2010 to June 2013 in a medical center. We identified the WICs and typed these conflicts according to the two foci (task content/process and interpersonal relationship) and the three properties (disagreement, interference, and negative emotion), and analyzed relevant data.

**Results:**

Of the 147 incidents with WIC, the most common related processes were patient transfer (20%), laboratory tests (17%), surgery (16%) and medical imaging (16%). All of the 147 incidents with WIC focused on task content or task process, but 41 (27.9%) also focused on the interpersonal relationship. We found disagreement, interference, and negative emotion in 91.2%, 88.4%, and 55.8% of the cases, respectively. Nurses (57%) were most often the reporting workers, while the most common encounter was the nurse-doctor interaction (33%), and the majority (67%) of the conflicts were experienced concurrently with the incidents. There was a significant difference in the distribution of worker job types between cases focused on the interpersonal relationship and those without (p = 0.0064). The doctors were more frequently as the reporter when the conflicts focused on the interpersonal relationship (34.1%) than not on it (17.0%). The distributions of worker job types were similar between those with and without negative emotion (p = 0.125).

**Conclusions:**

The institutional IRS is a useful place to report the workplace interpersonal conflicts actively. The healthcare systems need to improve the channels to communicate, manage and resolve these conflicts.

## Introduction

Interpersonal conflict is an important type of conflict [1] that a variety of its nomenclature exists, such as task, process, information, emotional and relationship conflicts [2–4]. This kind of conflict is often regarded as a negative term because individual interests are perceived to be opposed or negatively affected [5]. Recently, there have been concerns about workplace interpersonal conflict (WIC) and its impact on the healthcare system and the workers [6]. A variety of individuals, including doctors, nurses, co-workers, managers and administrative workers might have experienced conflicts [7]. The WICs were also commonly encountered in intensive care units [8], operating rooms [9] and emergency rooms [10, 11]. These high-risk health care areas often require intensive patient caring, timely decision-making, and multidisciplinary coordination among the workers [12]. WICs might be the consequence of poor communication [13], excessive work stress [14] or unsolved competing priorities of tasks [15]. Once developed, the WIC might, in turn, possess deleterious effects on the workers, such as interference with the team performance and reduction of staff satisfaction [16]. Furthermore, reports have associated WICs with lower-quality patient care, higher rates of medical errors, a higher level of staff burnout, and greater direct and indirect costs of care [17].

Since these consequences might compromise patient safety, we proposed that the workers of the healthcare system might report interpersonal conflicts in the process of patient care when they report patient safety events. Many healthcare systems worldwide have implemented the incident reporting system (IRS) to understand the occurrence of patient safety events [18]. Since 2000, our institution has established an IRS for the workers in the hospital to report safety-related events in the hospital. Previous reports of the studies on the WIC in the healthcare settings applied mainly questionnaire investigations and surveys [12, 19] while real-life case-specific descriptions of the WIC were lacking, probably because reporting directly from the workers might require an adequate channel. Whether or no the IRS might also be a possible channel for reporting the WIC had remained unclear.

## Materials and methods

### Study design and settings

This retrospective study was conducted at the National Taiwan University Hospital (NTUH) to analyze the collected data from the incident reporting system (IRS) of the institution from July 2010 to June 2013. The Research Ethical Committee A of the National Taiwan University Hospital approved the study and exempted the informed consents.

The NTUH was a 2,300-bed, university-affiliated medical center, containing about 6,400 full-time employees, which included more than 1,000 doctors and 2,700 nurses. In 2000, the hospital established the institutional IRS for safety-related events. It initially operated through a paper-based reporting process, and then adopted on-line reporting mechanism in 2005 by integrating into the hospital’s intranet. The reporting of the safety incident was voluntary and non-punitive, focused on safety-related events or concerns to the patients, workers and visitors. In addition to structured checkboxes for data entry, the online page also provided a free text field for the reporter to describe the events. After the reporting, the Center for Quality Management managed the data. The staff of this centralized unit of this institution verified the incidents, collected and analyzed pertinent information, performed important discussions with the workers from where the events occurred, and participated in the improvement activities.

The definition and severity of the safety-related incidents were classified as previously described in the literature [20–22]. Briefly, a safety incident or event is an unexpected or unintended event, which could have led to or did result in harm of the involved person. An adverse event was an injury caused during the health care process rather than by the underlying disease or condition of the individual. A no-harm event was an event, which resulted in no harm to the person, or the effect was minor that the individual could not even feel it. A near miss event was an event that may cause accident, injury or illness, but did not happen because of unintentional or timely intervention [20–22]. We excluded the reports from the database of IRS if the incidents were not related to any health care or service process for the patients, or not relevant to the health care environment provided for the care of the patients.

### Review of incidents and identification of workplace interpersonal conflicts

Our team of two doctors, three nurses and two quality managers from the Institution’s Center for Quality Management reviewed all available incident reports. All team members had at least one year’s training in the process. Four of the seven reviewers independently selected the reports they considered to contain the descriptions compatible with workforce conflict and recorded the type using the classification in Table 1 below. The three other members then participated in the assessment of selected reports and authenticated the consensus results. Records were de-identified and analyzed anonymously.

**Table 1:** Workplace Interpersonal Conflicts: Summary of Definitions*.

Property of the WIC	Focus of the WIC	
	Task Content or Task Process	Interpersonal Relationship
Disagreement	Disagreement with other about what should be done or how should be done in a task	Disagreement with the other’s personal values, views, preferences, etc.
Interference	Preventing the other from doing what they think should be done in a task or how a task should be done	Preventing the other from doing things unrelated to a task
Negative emotion	Anger and frustration directed to the other about what should be done in a task or how a task should be done	Anger and frustration directed to the other as a person

*Adopted from Barki and Harwick[4].

### Interpersonal conflict

We classified workplace conflicts using a modified Barki and Hartwick typology based scheme [4] (Table 1). We defined interpersonal conflict as “a dynamic process that occurs between interdependent individuals, groups, or both, as they experience negative emotional reactions to perceived disagreements and interference with the attainment of their goals.” The two types of focus of the interpersonal conflict identified included the conflicts related to task content or process, and the conflicts related to the interpersonal relationship. We explored each for disagreement, interference, and negative emotion as the property of the WICs. Disagreement reflects cognitive difference based on a divergence of values, needs, interests, opinions, or goals. Interference indicates conflict due to behavioral difference and used by one party to interfere with or oppose another party’s attaining its interests, objectives or goals. Such behaviors include debate, argumentation, competition, political maneuvering, backstabbing, aggression, hostility, and destruction. Negative emotions produce conflict and underlie fear, jealousy, anger, anxiety, and frustrations [4]. Table 1 depicts the typology of the classification of WICs used throughout this study. In each incident, the investigators identified at least one focus and property of conflict. Based on the text descriptions from the reporters and consensus after discussions, the investigators classified the incident-related processes that were related to the WICs and then identified the tasks that were considered most closely related to the occurrence of WICs.

### Collection of data

The investigators collected the following data for analysis: dates, times, place, and departments where the incidents and conflicts occurred, types and categories of the reported incidents, job types of the reporting workers and employees involved in the WIP, and working experience of the reporting workers. Records were de-identified and analyzed anonymously. We also included the data regarding the types of the conflicts into analysis together with the collected data.

### Statistical analysis

We first analyzed the health care characteristics relating to the development of WICs found in the reported incidents, such as health care related processes, worker types involved in the development of WIP, and severity of the reported incidents. We then described the typing of the WICs, based on the focus of the conflicts. We then further explore the possible association of job scenario and the development of WIP by comparing different characteristics among groups.

Results are summarized and expressed as counts and percentage for nominal variables, or median with range for ages. The chi-square test was used to compare across different categories, such as incident types, job types of the workers. The Mann-Whitney U-test was used to compare the age between groups of workers. Statistical analysis was performed using the SPSS 22 Software (SPSS Corp., Chicago, IL, USA). A P<0.05 was considered statistically significant.

## Results

During the 3-year study period, a total of 8,555 safety-related incidents (Table 2) were reported hospital-wide, with an average of 237 events every month. Among the 8,555 safety-related incidents, 147 (1.7%) had WIC. Strikingly none involved fall events and tubing/line events, and few involved medication. 96% involved health care or service processes (Table 3). Most commonly, they involved the transfer of patients between units or departments (20%), laboratory tests (17%), surgery (16%) and medical image examination and interventions (16%). For each category of the incident-related processes, the first tasks were the most frequent (64 incidents, 44%) in these processes associated with the conflict, such as the decision to start the process, the requesting for the care process and the scheduling for the process. (Table 3).

**Table 2:** Safety-related incidents during the study period.

Type	Number (%)
Fall	2,041 (23.9%)
Indwelling tubes and lines	1,577 (18.4%)
Medication	1,504 (17.6%)
Transfusion	827 (9.7%)
Diagnostic procedures	503 (5.9%)
General bedside care	184 (2.2%)
Self hurting	164 (1.9%)
Unexpected cardiopulmonary resuscitation	139 (1.6%)
Medical device	45 (0.5%)
Anesthesia	10 (0.1%)
Others	876 (10.2%)
Total	8,555

**Table 3:** Workplace interpersonal conflicts: Related processes and tasks (n = 147).

Incident-related process	Number (%)	Conflict-associated tasks in the process	Number (%)
Patient transfer	28 (20)	Decision on transfer	25 (17)
		Preparation for transfer	2 (1)
		Handoff	1 (1)
Laboratory tests	25 (17)	Test ordering	1 (1)
		Sample preparation	11 (7)
		Sample transporting	2 (1)
		Turnaround time	10 (7)
		Report maintenance	1 (1)
Surgery	24 (16)	Scheduling	10 (7)
		Preoperative preparation	5 (3)
		In-hospital transport	6 (4)
		Intraoperative care	1 (1)
		Postoperative care	1 (1)
		Operation note entry	1 (1)
Medical image examinations and interventions	23 (16)	Scheduling	9 (6)
		Preparation for examination	3 (2)
		In-hospital transport	6 (4)
		Continuity of care	2 (1)
		Patient identification	1 (1)
		Post-examination care	1 (1)
		Result reporting	1 (1)
Consultations	13 (9)	Request for consultation	10 (7)
		Equipment preparation	2 (1)
		Consultation response	1 (1)
Maintenance of facility and equipment	12 (8)	Supply turnaround time	5 (3)
		Facility & equipment maintenance	7 (5)
Transfusions	6 (4)	Request for transfusion	6 (4)
Medication	6 (4)	Medication order	1 (1)
		Dispensing	2 (1)
		In-hospital transport of medication	2 (1)
		Adverse reaction reporting	1 (1)
Resuscitations	3 (2)	Decision on resuscitation	1 (1)
		Cooperation during resuscitation	2 (1)
Outpatient clinic	3 (2)	Scheduling	2 (1)
		Medical record maintenance	1 (1)
Emergency Room Visit	1 (1)	Patient identification	1 (1)
Dialysis	1 (1)	Equipment maintenance	1 (1)
Other	2 (1)	Patient monitoring	1 (1)
		IT system maintenance	1 (1)

Table 4 summarizes the characteristics of the workers and working condition. Most commonly, the nurses were the reporter (57%), in a nurse-doctor encounter (33%). The WICs occurred most frequently during telephone communication (63%), at the same time when the incident developed (60%), during the daytime nursing shift (51%) (Table 4). We did not find any WIC in the same unit. The median age of the reporters was 7 years (range 0–31 years). The working experience was similar among different types of workers (p = 0.055), although the doctors tended to have less working years (median, 4 years; range, 0–31 years).

**Table 4:** Workplace interpersonal conflicts: Summary of 147 incidents.

Description	Number (%)
Total number of safety-related incidents with WIC	147 (100)
Reporting worker job type	
Nurse	84 (57)
Doctor	32 (22)
Other healthcare professional	24 (16)
Supporting department worker	7 (5)
Working experience of the incident reporter (year, mean, range)	9.3 (0.2–30.9)
Job types of the workers encountered in the WIC	
Nurse-doctor	48 (33)
Nurse-other healthcare professional	36 (24)
Doctor-doctor	18 (12)
Nurse-nurse	14 (10)
Nurse-supporting department worker	10 (7)
Doctor-other healthcare professional	6 (4)
Doctor-supporting department worker	5 (3)
Other encounters	10 (8)
Interaction and communication scenario of the WIC	
Face-to-face interaction	54 (37)
Telephone communication	93 (63)
Timing of the occurrence of conflict in relation to the incident	
Conflict occurred before the incident	4 (3)
Conflict occurred at the same time with the incident	89 (60)
Conflict occurred after the incident	54 (37)
Timing of the incident occurrence in relation to nursing shift	
Day shift	75 (51)
Evening shift	53 (36)
Night shift	19 (13)

**Fig 1:**
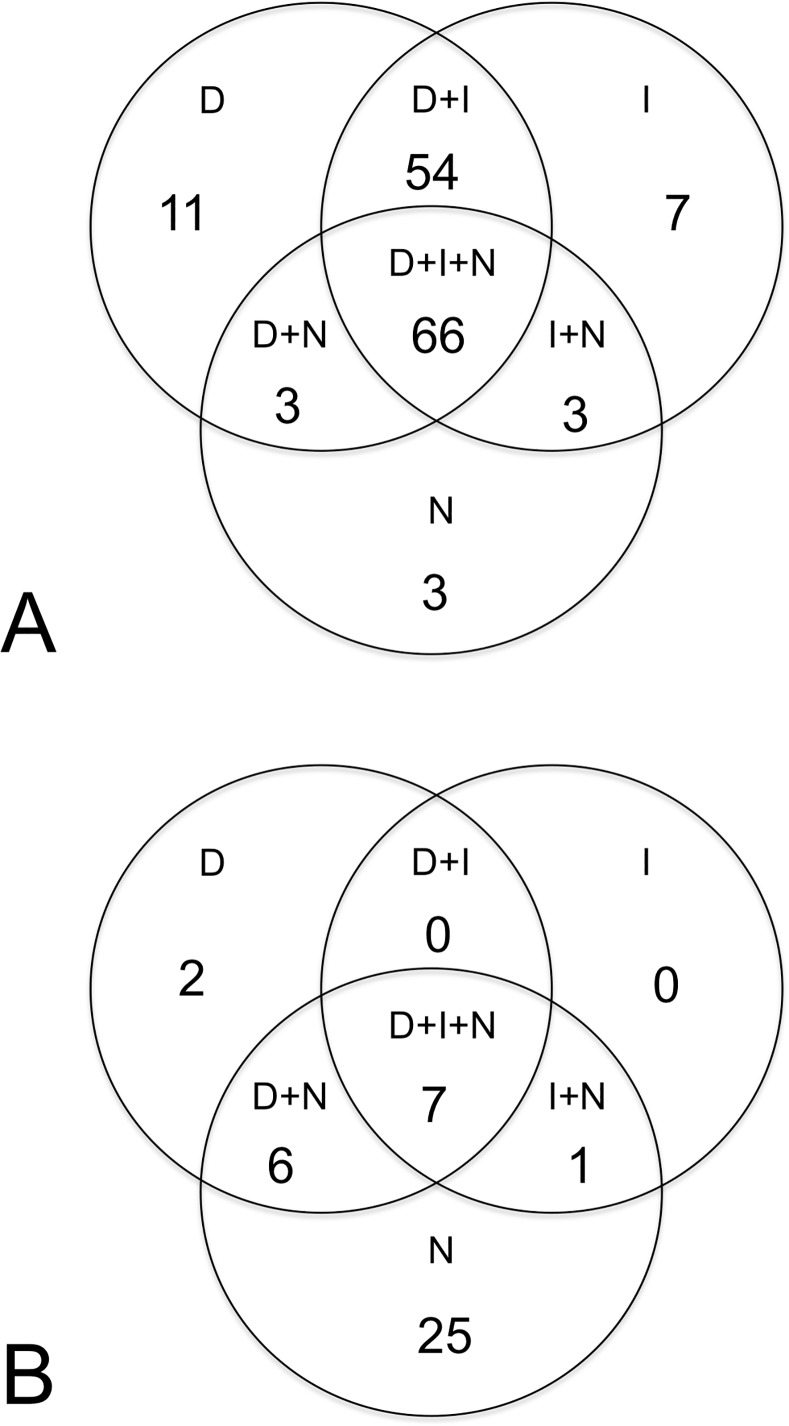
Venn diagrams of the workplace interpersonal conflicts. (A) Conflicts focused on the task process/task content. (B) Conflicts focused on the interpersonal relationship. D = disagreement; I = interference; N = negative emotion.

Table 5 shows the types of WIC. All of the 147 incidents with WIC focused on task content or task process, but 41 (27.9%) also focused on the interpersonal relationship (Table 5). Fig 1 shows the Venn diagram to indicate different combinations of WIC properties in these incidents. Most (85.7%) of the cases focused on the task content or task process had a mixed property of conflicts. This included especially the “disagreement + interference + negative emotion” combination (66 cases, 44.9%) and the “disagreement + interference” combination (54 cases, 36.7%) (Fig 1A). On the contrary, the majority (25 cases, 61.0%) the WIC focusing on the interpersonal relationship had a single property of negative emotion without any description of disagreement or interference (Fig 1B). For all of the 147 cases, 82 (55.8%) had a negative emotion. These included 42 (28.6%) focused on task content or process, 7 (4.8%) on the interpersonal relationship, and 33 (22.4%) on both. Table 6 shows examples of reporting descriptions from the workers considered as having the WIC in different categories.

**Table 5:** Workplace interpersonal conflicts: Types of the 147 incidents.

Property of the WIC	Focus of the WIC		
	Task Content or Task Process (147 [100%])	Interpersonal Relationship (41 [27.9%])	Either Focus Combined Number (147 [100%])
Disagreement	134 (91.2%)	16 (10.9%)	134 (91.2%)
Interference	130 (88.4%)	8 (5.4%)	130 (88.4%)
Negative emotion	75 (51.0%)	40 (27.2%)	82 (55.8%)

**Table 6:** Workplace interpersonal conflicts: Examples.

Focus Category	Focus Property	Summaries of Text Descriptions*
Task content or task process	Disagreement	● The nurse reported that the lab technician insisted on not having received the sample, but the nurse had checked the tracking system and was sure that they had submitted the sample. ● The nurse reported that the patient should not stay at the emergency room because the patient should be transported directly to the ICU. ● The technician said that the doctors should not cancel the order of blood transfusion by telephone despite knowing that they might be busy in the Operation Room.
	Interference	● The nurses transported the patient to the general ward, but on arrival, they were asked to transport this patient to the operation room without confirmed message. ● The anesthesiologist was unable to key in assessment data to the electronic record system because the requesting department did not establish an eligible patient list. ● The doctor asked that the nurse should stay at the bedside during the examination, which was declined by the nurse.
	Negative emotion	● The nurses expressed their anger about the operation schedule that resulted in prolonged waiting for the patients. ● The nurse was surprised and upset about that the staff transported the patient to the ICU before confirmation of the transfer timing. ● The nurse expressed a very frustrating situation to wait for such a long time for gathering all of the team members to transport the patient for radiologic examination and intervention.
Interpersonal relationship	Disagreement	● The nurse at the ICU received the comments from a doctor unrelated to the care team for the indicated patient and disagreed with the comment by stating that the doctor had no right to intervene the care for that patient. ● The nurse reported that they did not need to perform the job they are not supposed to do, such as explain to the patients about the operation procedure. ● The nurse reported that the way the indicated doctor spoke to the nurse was not acceptable.
	Interference	● The doctor asked the staff to do more works that another discipline should do, which bothered the nurses. ● A doctor who was unrelated to the care team for the patient tried to put opinions into the patient care. ● The doctor unrelated to the Emergency Room and the patient care team asked the Emergency Room staff to admit the patient to the ward.
	Negative emotion	● The nurse reported her staff members were very upset about the poor attitude and interaction from the transferring unit. ● The lab technician stated the frustration and lack of respect during the contact with nurses for laboratory sample issue. ● The doctor expressed his anger about the nurse repeatedly asked him to correct the medication order without confirmation.

*Translated from the original Chinese texts.

As shown in Table 7, there was a significant difference in the distribution of worker job types between cases with or without a focus on the interpersonal relationship (p = 0.0064). Nurses accounted for a lower proportion of the reporters of cases with interpersonal relationship than the cases without this type of conflict (34.1% vs. 66.0%). However, cases with interpersonal relationship had more doctors involved than those without this conflict (34.1% vs. 17.0%). There working experience of the employee who reported interpersonal relationship (n = 41; median, 6 years; range, 1–31 years) was similar to those who did not (n = 106) (median, 8 years; range, 0–31 years) (p = 0.397).

**Table 7:** Personnel involved and interpersonal relationship.

Worker job type	Conflicts focused on the interpersonal relationship		
	Present (n = 41)	Absent (n = 106)	*p*-value
Reporter of the conflict in the incident			
Doctor	14 (34.1%)	18 (17.0%)	0.0064
Nurse	14 (34.1%)	70 (66.0%)	
Other healthcare professions	10 (24.4%)	14 (13.2%)	
Supporting worker	3 (7.3%)	4 (3.8%)	
Doctor reportedly involved in the conflict			
Yes	27 (65.9%)	51 (48.1%)	0.053
No	14 (34.1%)	55 (51.9%)	

In the 82 incidents with negative emotion, 45 (54.9%) were described by the reporters, while 15 (18.3%) by the counterpart workers and 22 (26.8%) by both sides involved in the conflict. Also, 26 (31.7%) described disruptive languages whereas 9 (11.0%) had disruptive physical behaviors. Although the job types were similar between incidents with negative emotion and those without (p = 0.125), there were more doctors as the reporters for WIC in cases with a negative emotion than the cases without (28.0% vs. 13.8%) (Table 8). This result was in contrast to the finding that there were fewer nurses as the reporter in the case with a negative emotion (50.0% vs. 66.2%). There was no difference of work experience between the employee who reported negative emotions (n = 82; median, 5 years; range, 1–25 years) and who did not (n = 65) (median, 8 years; range, 0–31 years) (p = 0.265).

**Table 8:** Personnel involved and negative emotion.

Worker type	Negative emotion in the report		
	Present (n = 82)	Absent (n = 65)	*p*-value
Reporter of incidents			
Doctor	23 (28.0%)	9 (13.8%)	0.125
Nurse	41 (50.0%)	43 (66.2%)	
Other healthcare professions	13 (15.9%)	11 (16.9%)	
Supporting worker	5 (6.1%)	2 (3.1%)	

Table 9 summarizes the comparison of the distributions of incident types based on the severity of outcome between the two groups that contained (n = 147) or did not contain (n = 8408) WIC, which was statistically different (P<0.0001). The incidents with WIC had less harm to the patients than those without reported conflicts (Table 9). None of the patients associated with the events containing WIC died in the hospital; this was in contrast with that 18 patients linked to the incidents that did not have WIC died at hospital discharge.

**Table 9:** Workplace interpersonal conflicts and severities of incidents.

Severity	Incidents without reported WIC (n = 8408)	Incidents with reported WIC (n = 147)	*P* value
According to event type			
Near miss	1184 (14.1)	24 (16.3)	< 0.0001
No harm event	4660 (55.4)	107 (72.8)	
Mild adverse event	2256 (26.8)	10 (6.8)	
Moderate adverse event	270 (3.2)	5 (3.4)	
Severe adverse event	20 (0.2)	0 (0)	
Very severe adverse event	18 (0.2)	1 (0.7)	

## Discussion

The main finding of this study was that the employee of the hospital applied the incident reporting system (IRS) to actively report the workplace interpersonal conflicts (WICs) although the IRS was originally designed for the reporting of safety events.

Our study provided the evidence of WIC as the dedicated workers of the institution described the scenarios and encounters related to the conflicts in the incident reporting system. Although we identified only 1.72% of the reported safety incidents as having the WIC, there might be a high probability of underestimation. One of the explanations is that the institution originally designed the IRS for the reporting of safety incidents that the descriptions by the reporters focused mainly on the events rather than the conflicts. Moreover, these WICs were the interpersonal conflicts between people of different units. Individuals in the same unit or department might report the conflicts to the same one supervisor, rather than to the IRS. In fact, we did not find an intra-unit WIC in this study. Also, the culture of the healthcare environment, as well as the social background, might also influence the reporting of WICs. In some cultural contexts, the organization might not encourage the reporting of conflicts because of the emphasis on harmony to avoid conflict[23]. Nevertheless, we suggest the healthcare system should promote the reporting of the conflicts. Other authors also suggested that the organizations asked their people to discuss conflicts openly and productively to strengthen the interpersonal relationships [24].

Traditionally, interpersonal conflicts among nurses were called ‘horizontal violence’[25]. However, we found a substantial number of WICs focusing on interpersonal relationship across different disciplines and units. This finding suggests that horizontal violence might be more common than previously perceived. Although researchers reported that newly graduated in the first year of their practice might encounter horizontal violence [25], we showed that the occurrence of WICs focusing on the interpersonal relationship was not related to working experience. Therefore the healthcare organization might need to pay attention to the possibility of horizontal violence in workers with any level of working experience.

Because all of the WICs in this study focused on the task content or task process, we suggest the need for encouraging the conflict reporting for in the improvement of the process of health care. From the teamwork’s point of view, goal orientation moderates the relationship between conflict and team performance [26]. Therefore, while the workers report conflicts, they should have the mutual understanding on the goal of care, and communication undoubtedly plays a vital role in the management of conflicts. However, adequate communication is warranted, since too much interaction might also contribute to misunderstanding because of perceived words, body language, and expressions lead to intent [5]. For situations that effective communication might not be feasible during the care process, reporting WICs focusing on task content and task process through the IRS might be seen as a form of moderate way of communication to avoid more conflicts, as the reporter had perceived. Since many WICs also focused on the interpersonal relationship, we also suggest that these types of reports should be deemed as an internal informal complaint process [27]. Conflicts in the interpersonal relationship might negatively affect patient care by interfering with one’s ability to work with the other members of the health care team [28], especially when there is disruptive behavior [29]. In a survey, more than 50% of healthcare workers witnessed the disruptive behaviors, and 18% reported that they were aware of a particular adverse event that occurred as a direct result [30]. For a better working environment, the organization should encourage the healthcare workers to report the conflicts.

The use of IRS as a channel for reporting interpersonal conflict might have the potential of providing a chance to systemic improvement. Previous reports suggesting controversial conclusions about the benefits of conflicts and performance and employee satisfaction [16, 31–35]. The application of formal reporting system such as IRS might provide the chance to improve interpersonal conflicts. Management of the reported events are mainly task-oriented, therefore might reduce the tension between the workers with interpersonal conflicts and focus more on the goal of the task and the expected provided care.

Our study has several limitations. First, since this study was retrospective and the IRS of this hospital did not provide a structured form for reporting WICs, we would miss a proportion of WICs experienced by the healthcare workers who encountered the incidents. It was also difficult to validate these WICs mainly based on the report contents; this was in contrast to the reports of incidents, which might be followed by further investigations as indicated. Second, we did not know the presence of WICs during the patient care processes if there was no incident reported. Although the establishment of a formal process for internal complaints might be necessary, we believe that at least some of the WICs would be considered not subjected for a formal reporting as complaints. Third, we did not investigate how the workers coped with WICs. Researchers had suggested a variety of types of behaviors for managing conflicts in the context of “conflict management strategies” (i.e. a repertoire of reactions to a conflict that individuals may adopt depending on the situations) or “conflict management styles” (implying a fixed tendency) [36]. Furthermore, approaches to managing conflict in organizations have been suggested [37]. Understand the conflict-managing behaviors of the workers was beyond the scope of this study and might require other approaches in addition to the incident report system here we used.

In conclusion, the institutional incident reporting system is a useful place to actively report the workplace interpersonal conflicts (WICs) related to task content and task process, and interpersonal relationship. The healthcare systems need to improve the channels to communicate, manage and resolve interpersonal conflicts.

## Supporting information

S1 Dataset Case File. The case data related to the workplace interpersonal conflicts retrieved from the incident reporting system of the institution.
(XLSX)
